# Gray Matter Volume Changes in the Apathetic Elderly

**DOI:** 10.3389/fnhum.2015.00318

**Published:** 2015-06-02

**Authors:** Hongjie Yan, Keiichi Onoda, Shuhei Yamaguchi

**Affiliations:** ^1^Department of Neurology, Faculty of Medicine, Shimane University, Izumo, Japan

**Keywords:** apathy, voxel-based morphometry, gray matter volume, basal ganglia, frontal lobe, precentral gyrus

## Abstract

This study is to test the hypothesis that apathy in healthy participants is closely related to the prefrontal-basal-ganglia circuit and associated structural changes. We selected 36 healthy aged participants with (*n* = 18) or without apathy (*n* = 18) from our database. Participants underwent structural MRI scanning, providing data for voxel-based morphometric analysis to explore gray matter changes associated with apathy. Compared to the non-apathy group, the apathy group showed reduced gray matter volume of the right putamen, whereas volumes of the bilateral inferior frontal gyri and left inferior occipital gyrus showed increase. When depression scores were included in a regression model as a covariate, apathetic participants showed decreased gray matter volume in the right precentral gyrus compared to the non-apathetic participants. These findings suggest that apathy is associated with the gray matter volume in the prefrontal-basal-ganglia network, and may have a neuroanatomical basis distinct from depression in healthy elderly.

## Introduction

Apathy is defined as a lack of motivation that is not caused by a disturbance in consciousness, cognitive impairment, or emotional distress (Marin et al., 1991), and this symptom can cause dysfunctions in elaboration, execution and management of goal-directed behaviors (Brown and Pluck, 2000). Nowadays, apathy is recognized as a frequent neuropsychiatric symptom not only in neurodegenerative diseases, such as Parkinson’s disease (Dujardin et al., 2007), Huntington’s disease (Di Maio et al., 1993), and progressive supranuclear palsy (Litvan et al., 1996), but also in stroke (Starkstein et al., 1993), dementia (Kuzis et al., 1999), and brain injury (Diaz et al., 2012). Studies have shown that apathy does influence patients’ quality of life (Yeager and Hyer, 2008) and recovery from illness (Politis et al., 2004). Although the exact mechanism is still not clear, the prefrontal-basal-ganglia system is thought to play a key role in apathy (Levy and Dubois, 2006). Most lesion studies have identified the prefrontal cortex and basal ganglia as target regions responsible for apathy symptoms. Apathy symptoms are observed after direct lesions of prefrontal cortex (Eslinger and Damasio, 1985; Stuss et al., 2000), and they can also appear indirectly in many diseases that are accompanied by lesions in the basal-ganglia (Laplane et al., 1989; Engelborghs et al., 2000). For instance, auto-activation deficit was observed in PSP, in which the basal-ganglia dysfunction caused the prefrontal hypometabolism (D’Antona et al., 1985; Baron, 1994). These findings suggest that apathy is associated with disruption of the network involving the frontal lobe and basal ganglia (Middleton and Strick, 2000; Kimura et al., 2003).

Apathy can occur not only in patient populations but also in healthy individuals free of obvious pathology. However, only one study has assessed the structural changes associated with apathy in healthy participants free of any related brain diseases (Grool et al., 2014). In that study, gray matter volume reductions in the frontal and temporal lobes as well as the thalamus were related to apathy symptoms, but no significant changes in the basal ganglia were noted. One reason for this might be that they assessed apathy symptoms using only three items from a geriatric depression scale. Apathy symptoms should be assessed by more elaborate instruments that have been specifically developed for apathy assessment. In this study, we assessed apathy using the apathy scale originally developed by Starkstein et al. (1992) and subsequently modified for use with the Japanese population (Okada et al., 1997). Here, we studied whether apathetic healthy subjects showed any structural changes in the brain including within the prefrontal-basal-ganglia system.

## Materials and Methods

### Participants

The participants were selected from among the individuals who participated in health screening at the Shimane Institute of Health Science from 2007 to 2013. The database consisted of 445 subjects. All participants provided informed consent, and the medical ethics committee of Shimane University approved the study protocol. The health check included physical examination, detailed medical history (disease, life habits, medication, and treatment), laboratory blood tests, neuropsychological assessment, and head MRI. The subjects included in this study were required to have no history of neurologic or psychiatric conditions such as cerebrovascular disease, dementia, depression, or other psychiatric illnesses. The age was from 60 to 70 years old. The MRI did not show any brain lesions including silent brain lesions, silent brain infarctions, pathological white matter lesions, and microbleeds.

Neuropsychological assessments included the following tests: the mini-mental state examination (MMSE) of general cognitive ability (Folstein et al., 1975), subtests of the Okabe intelligence test, which is a modified and simplified version of the Wechsler adult intelligence scale, including tests assessing information, mental control, digit span, and paired associate and non-associate learning (Kobayashi et al., 1987), and Kohs’ block-design test (Dureman and Sälde, 1959) for visuospatial ability. For assessing frontal lobe executive function we used the frontal assessment battery (FAB) (Dubois et al., 2000), Kana-pickup test (Kaneko, 1990), and verbal fluency test (Ruff et al., 1996). Depression was also assessed using the Japanese version of Zung’s self-rating depression scale (SDS) (Zung, 1965; Fukuda and Kobayashi, 1973). For all included participants, cognitive function test scores were within the normal range, i.e., MMSE score >26, FAB score >12, and Okabe score >35. Apathy was assessed using the apathy scale, which was originally developed by Starkstein et al. (1992) and modified for use with Japanese individuals (Okada et al., 1997). The division score on the apathy scale was 16 points. This division score was chosen as appropriate using our previous study on Japanese stroke patients (Okada et al., 1997). Ultimately, we selected 36 subjects (19 males and 17 females). Eighteen of these had a score on the apathy scale between 16 and 23 and made up the apathy group. Another eighteen had a score between 0 and 5 and comprised the non-apathy group. Statistics about each group’s general information and their neuropsychological examination results are given in Table 1.

**Table 1:** **Demographic characteristics and neuropsychiatric test scores**.

	A (*n* = 18) Mean (SD)	NA (*n* = 18) Mean (SD)	*p*-value
Age (years)	63.7 (3.0)	64.8 (3.0)	0.28
Gender (male/female)	10∕8	9∕9	0.74^a^
Years of education	12.7 (2.7)	13.2 (2.4)	0.61
Mini-mental state examination	28.3 (1.7)	28.9 (1.4)	0.30
Okabe test	42.3 (6.6)	45.8 (4.9)	0.09
Koh’s block-design test	99.1 (19.9)	105.4 (13.9)	0.28
Frontal assessment battery	15.9 (1.8)	16.4 (1.3)	0.31
Kana-pickup test	40.2 (9.2)	43.6 (13.3)	0.38
Verbal fluency test	7.8 (3.3)	10.4 (4.6)	0.07
Zung’s self-rating depression scale	38.6 (5.0)	28.2 (5.1)	0.0001
Apathy scale	19.2 (2.1)	2.6 (1.7)	0.0001

*A, apathy group; NA, non-apathy group*.

*^a^Chi-square test*.

*Other *p*-values are from *t*-test results*.

### MRI image acquisition

Scans were performed using a 1.5-T MRI (Symphony, Siemens) at the Shimane Institute of Health Science. Whole brain 3D T1-weighted images (T1WI) were obtained with the following parameters: repetition time (TR) = 1960 ms, echo time (TE) = 3.68 ms, matrix = 256 × 256, field of view (FOV) = 220 mm × 220 mm, voxel size = 1.5 mm × 1.5 mm × 1.5 mm, flip angle = 15°, slice thickness = 1.05 mm, and 120 slices without gap.

### Voxel-based morphometry

Data analysis was conducted using Statistical Parametric Mapping (SPM) software, Version 8 for Windows (SPM8, http://www.fil.ion.ucl.ac.uk/spm/) and Data Processing Assistant for Resting-State fMRI (Yan and Zang, 2010). Both programs work under MATLAB (MathWorks, Natick, MA, USA). The statistical image analysis was performed using SPM8, and DPARSF software was used for VBM (voxel-based morphometry) analysis. The following five steps were employed: (1) manual reorientation of the images to the center point of the anterior commissure (Friston et al., 1989). (2) The MR images were segmented into three parts: gray matter, while matter, and cerebrospinal fluid using the standard unified segmentation model in SPM8 (Ashburner and Friston, 2005). (3) The gray matter templates were then created using diffeomorphic anatomical registration through exponentiated lie algebra (DARTEL), an improved VBM analysis method, which can achieve more accurate inter-subject registration, realignment of small deformations, and better spatial normalization (Ashburner, 2007). (4) After an initial affine registration of the gray matter DARTEL templates to the tissue probability maps in the Montreal Neurological Institute space was implemented, the non-liner warping of gray matter images was performed to the template with 1.5-mm cubic resolution. (5) Images were then modulated to ensure the relative volumes of gray matter were preserved following the spatial normalization procedure. (6) The modulated and normalized gray matter images were smoothed using a 8-mm full-width-at-half-maximum Gaussian kernel.

### Statistical analysis

Differences in gray matter volume over the whole brain between the two groups were tested in SPM8 using one-way analysis of covariance (ANCOVA), using age and gender as covariates of no interest. A comparison of the two groups using SDS scores as a covariate of no interest was also performed. The significance threshold was set to *p* < 0.001 with more than 100 voxels in every cluster. Further statistical analysis was carried out using IBM SPSS statistics 21 (IBM SPSS Inc., Chicago, IL, USA), including Chi-square and independent samples *t*-tests on participant characteristics, where the significance threshold was set to *p* < 0.05.

## Results

There were no significant differences between the apathy and non-apathy groups in terms of age, gender, years of education, and neuropsychological test scores. The apathy group had higher depression scores compared to the non-apathy group (Table 1). Apathy scores were significantly correlated with depression scores (*r* = 0.58, *p* < 0.001).

**Figure 1:**
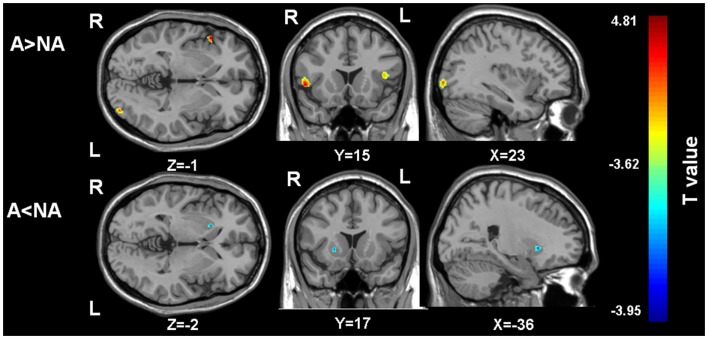
**The regions showing gray matter volume differences between the two groups**. A, apathy group; NA, non-apathy group. (Threshold at *p* uncorrected <0.001 and voxels more than 100). A > NA: the regions showing larger gray matter volumes in the apathy group compared to the non-apathy group. A < NA: the regions showing larger gray matter volumes in the apathy group compared to the non-apathy group.

**Figure 2:**
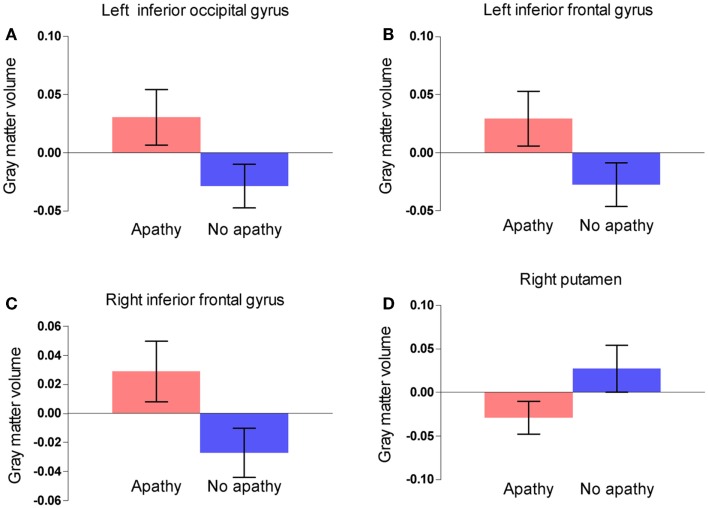
**Comparisons of gray matter volume within the clusters between two groups in the left inferior occipital gyrus (A), the right inferior frontal gyrus (B), the left inferior frontal gyrus (C), and the right putamen (D)**. Error bars show 95% confidence intervals.

Gray matter volumes were compared between the two groups via a whole brain analysis (Table 2). Significant group differences in the gray matter volume were observed at two clusters in the frontal lobe, one cluster in the occipital lobe, and one cluster in the basal ganglia. Gray matter volume in the right putamen was significantly smaller in the apathy group (Figure 1). On the other hand, gray matter volumes in the inferior frontal gyrus of both hemispheres as well as the left inferior occipital gyrus were actually larger in the apathy group compared to the non-apathy group. Figure 2 shows direct comparisons for gray matter volumes of each region between the two groups (Figure 2).

**Table 2:** **Whole brain analysis comparisons of gray matter volume between apathy and non-apathy groups**.

Group comparison	Region (peak of cluster)	Cluster size	Cluster-level *p*-value	MNI coordinates of the peak voxel (*x*, *y*, *z*)	*T* value of peak voxel
A > NA	Left inferior frontal gyrus	143	<0.001	−42, 17, 10	4.11
	Right inferior frontal gyrus	219	<0.001	53, 18, 0	4.81
	Left inferior occipital gyrus	160	<0.001	−36, −91, −2	4.27
A < NA	Right putamen	104	<0.001	23, 17, −3	3.80

**p*-values are uncorrected with voxels more than 100*.

*A, apathy group; NA, non-apathy group*.

**Figure 3:**
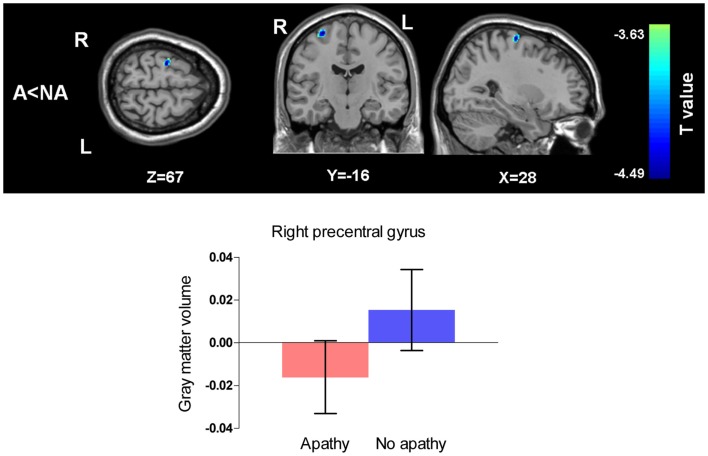
**Comparison of gray matter volumes between two groups with SDS scores as a covariate of no interest**. (Threshold at *p* uncorrected <0.001 and voxels more than 100). A, apathy group; NA, non-apathy group. Apathy group had decreased gray matter volume in the right precentral gyrus compared with non-apathy group. Error bars show 95% confidence intervals.

When the effects of depressive symptoms were adjusted for SDS scores as a confounding variable and the *p*-value was set as <0.005 uncorrected, the clusters mentioned above still existed though the number of voxels in the clusters decreased. When we used the same statistical criteria (*p* < 0.001 with voxel number more than 100), the apathy group showed only significantly smaller gray matter volume in the right precentral gyrus compared to the non-apathy group (Figure 3).

## Discussion

The aim of this study was to explore structural brain changes in apathetic healthy participants, particularly in the regions of the prefrontal-basal-ganglia circuit on the basis of the findings in previous lesion studies. We found that gray matter volume changed in three regions within the prefrontal-basal-ganglia circuit in association with apathy symptoms. These findings are in line with the role of this network known to play in motivational functioning. In addition, volume change in the occipital region was also related to apathy. In our apathy group, there were five subjects who also had symptoms of being slightly suspected depressive, while the SDS scores of the rest of people in both groups were in the normal range. (The severity of depression was divided into three levels in our database study, 0–39 normal, 40–59 suspected depressive, and 60–80 possible to be depression.) Since SDS score and apathy score are often correlated with each other, some reports have illustrated that apathy and depression share similar brain mechanisms (Marin et al., 1993; Starkstein et al., 1993). Thus, the regions identified in the first analysis might be partially associated with depression symptoms in addition to apathy symptoms. In the second analysis, on the other hand, we found significant volume reduction in the right precentral gyrus in the apathy group when co-occurring depressive symptoms were controlled. This finding is in line with several lesion and functional studies showing that apathy is a symptom coexisting with depression but it can also occur dissociatively (Levy et al., 1998; Andersson et al., 1999; Alexopoulos et al., 2013).

Reduction in gray matter volumes for several structures, including the nucleus accumbens, right posterior cingulate gyrus, and bilateral inferior frontal gyrus, were reported in apathetic patients with Parkinson’s disease (Kostić and Filippi, 2011; Carriere et al., 2014). Apathetic amyotrophic lateral sclerosis patients also show significant gray matter volume reductions in the occipital lobe and inferior frontal gyrus (Tsujimoto et al., 2011). These findings for neurological diseases are intriguing because the patterns of structural changes associated with apathy are in part opposite to our pattern for healthy participants. These discrepancies suggest that neurodegenerative disease processes in the brain are important for investigating the relationship between neuropsychological symptoms and their anatomical basis. In healthy participants, it may be possible that morphological compensation (i.e., volume increase) occurs in the regions responsible for apathy symptoms. Apathetic symptoms are typically associated with slowed and inefficient processing and integration of information; for example, the interaction between the inferior frontal gyrus and inferior occipital gyrus is mediated by the inferior fronto-occipital fasciculus (Martino et al., 2010). The structural changes in these two cortical regions found in our study might be compensations that enable effective behavior execution in healthy participants. Actually, most of our subjects were still at work or engaged in social activities. The compensatory hypothesis is worthy of examination in future studies. In addition, apathy can be associated with a variety of factors in healthy elderly, such as low income, low instrumental activities of daily living, or cognitive impairment (Adams, 2001; Onyike et al., 2007). Apathy in our healthy subjects may be related to some cognitive impairment because the verbal fluency score was slightly lower in the apathy group although the MMSE score was comparable between the two groups.

On the other hand, volume reduction of the basal ganglia was associated with apathy in healthy participants, as seen in patients with various neuropsychiatric diseases. This is in line with reductions of blood flow and metabolism in the basal ganglia, which is reported in patients with apathy after stroke and Parkinson’s disease (Okada et al., 1997; Isella et al., 2002; Onoda et al., 2010). The putamen is related to action-reward association learning and storage of motor memories (Balleine et al., 2007). When lesions occur in the putamen, they cause disturbances in motor initiation (Miller and Cummings, 2007). Volume reduction in the putamen seems to play a critical role in the appearance of apathy in healthy participants, too. In addition, the inferior frontal gyrus plays a general role in both executive function and inhibitory control (Swick et al., 2008; Zheng et al., 2008; Hampshire et al., 2010). Thus, the increased volume of the inferior frontal gyrus observed here may also contribute to augmented psychomotor or psychosocial inhibition observed in apathetic participants.

Apathy and depression are associated with structural brain changes in several pathological conditions, including Alzheimer’s disease, mild cognitive impairment, and major depression (Marin et al., 1994; Lavretsky et al., 2008). The above mentioned structural changes associated with apathy might be confounded by depression. We found that apathy was associated with the gray matter volume reduction in the right precentral gyrus after adjustment for depressive symptoms. In a previous VBM study, the atrophy in bilateral precentral gyrus has been reported in apathetic patients with Parkinson’s disease, and the high-apathy scores were correlated with low gray matter density (Reijnders et al., 2010). From the point of view of regional cerebral blood flow (rCBF), Kang’s study on AD patients reported that compared with non-apathy group, apathy group showed lower rCBF in bilateral precentral gyrus (Kang et al., 2012). Although depression group also demonstrated the comparable rCBF reduction in the precentral gyrus, the extent of regions with low rCBF in apathy group was larger than depression group, which partly supports our results. In a task-based study of planning performance in patients with schizophrenia, the precentral gyrus of patients with high level of apathy showed lower task-related activation than healthy control (Liemburg et al., 2015). Thus, our preliminary results expand the previous studies and are in line with the notion that apathy and depression have a different neuroanatomical basis. This point was already reported by previous studies in neurodegenerative diseases (Holthoff et al., 2005; Kirsch-Darrow et al., 2006; Naarding et al., 2009) while we got the same view in healthy elderly.

The limitations of our study are its cross-sectional design and relatively small number of subjects. Long-term follow-up studies with a larger number of participants would more confidently explore the causal relationship between brain volume change and apathy. Even though there may be some limitations, the current study identified structural changes associated with well-defined apathy in healthy participants, and the findings contribute to the understanding of the brain mechanisms underlying apathy independent of pathological changes occurring in neuropsychiatric diseases. It is also known that apathy is a prodromal symptom in many degenerative neurological disorders (Delrieu et al., 2014; Mollenhauer, 2015). The current study warrants future study to elucidate the distinctive pathophysiology of apathetic symptoms in patients with brain pathology. In addition, the identified brain structures that changed with apathy, specifically the precentral gyrus, could be plausible targets for therapeutic interventions targeting apathy; for example, treatment by means of repetitive transcranial magnetic stimulation (Oguro et al., 2014). Furthermore, analysis of functional connectivity among brain regions may complement the current findings in the future.

## Author Contributions

Conceived and designed the experiments: HY, KO, and SY. Performed the experiments: HY, KO, and SY. Analyzed the data: HY. Contributed reagents/materials/analysis tools: HY, KO, and SY. Wrote the paper including drafting and revising it: HY, KO, and SY.

## Conflict of Interest Statement

The authors declare that the research was conducted in the absence of any commercial or financial relationships that could be construed as a potential conflict of interest.
